# Electrophysiological Signatures of Visual Recognition Memory across All Layers of Mouse V1

**DOI:** 10.1523/JNEUROSCI.0090-23.2023

**Published:** 2023-11-01

**Authors:** Dustin J. Hayden, Peter S. B. Finnie, Aurore Thomazeau, Alyssa Y. Li, Samuel F. Cooke, Mark F. Bear

**Affiliations:** ^1^The Picower Institute for Learning and Memory, Department of Brain and Cognitive Sciences, Massachusetts Institute of Technology, Cambridge, Massachusetts 02139; ^2^Biochemistry Program, Wellesley College, Wellesley, Massachusetts 02481

**Keywords:** long-term habituation, novelty detection, primary visual cortex, SRP, stimulus-selective response potentiation, visual recognition memory

## Abstract

In mouse primary visual cortex (V1), familiar stimuli evoke significantly altered responses when compared with novel stimuli. This stimulus-selective response plasticity (SRP) was described originally as an increase in the magnitude of visual evoked potentials (VEPs) elicited in layer 4 (L4) by familiar phase-reversing grating stimuli. SRP is dependent on NMDA receptors (NMDARs) and has been hypothesized to reflect potentiation of thalamocortical (TC) synapses in L4. However, recent evidence indicates that the synaptic modifications that manifest as SRP do not occur on L4 principal cells. To shed light on where and how SRP is induced and expressed in male and female mice, the present study had three related aims: (1) to confirm that NMDAR are required specifically in glutamatergic principal neurons of V1, (2) to investigate the consequences of deleting NMDAR specifically in L6, and (3) to use translaminar electrophysiological recordings to characterize SRP expression in different layers of V1. We find that knock-out (KO) of NMDAR in L6 principal neurons disrupts SRP. Current-source density (CSD) analysis of the VEP depth profile shows augmentation of short latency current sinks in layers 3, 4, and 6 in response to phase reversals of familiar stimuli. Multiunit recordings demonstrate that increased peak firing occurs in response to phase reversals of familiar stimuli across all layers, but that activity between phase reversals is suppressed. Together, these data reveal important aspects of the underlying phenomenology of SRP and generate new hypotheses for the expression of experience-dependent plasticity in V1.

**SIGNIFICANCE STATEMENT** Repeated exposure to stimuli that portend neither reward nor punishment leads to behavioral habituation, enabling organisms to dedicate attention to novel or otherwise significant features of the environment. The neural basis of this process, which is so often dysregulated in neurologic and psychiatric disorders, remains poorly understood. Learning and memory of stimulus familiarity can be studied in mouse visual cortex by measuring electrophysiological responses to simple phase-reversing grating stimuli. The current study advances knowledge of this process by documenting changes in visual evoked potentials (VEPs), neuronal spiking activity, and oscillations in the local field potentials (LFPs) across all layers of mouse visual cortex. In addition, we identify a key contribution of a specific population of neurons in layer 6 (L6) of visual cortex.

## Introduction

Passive exposure to innocuous sensory stimuli that do not reliably predict reward or punishment produces habituation of innate behavioral responses across a wide range of organisms (Thompson and Spencer, 1966; Rankin et al., 2009). This conservation of function reflects the fundamental importance of habituation, which putatively serves to reduce energy use and enable the allocation of attention toward salient elements of the environment. Habituation is accompanied by a range of effects on evoked neural activity in different model organisms (Grill-Spector et al., 2006; Cooke and Ramaswami, 2020), yet we do not yet have a clear picture of how this foundational form of learning is implemented within the mammalian brain. In a paradigm we have previously developed, head-restrained mice briefly exposed to an identical phase-reversing, oriented sinusoidal grating stimulus over successive days exhibit behavioral habituation that is accompanied by pronounced stimulus-selective response plasticity (SRP) in binocular primary visual cortex (V1). Specifically, visual-evoked potentials (VEPs) recorded in layer 4 (L4) elicited by phase reversals of these progressively familiar stimuli undergo a significant potentiation across days. Multiple molecular manipulations of V1 disrupt both SRP and accompanying behavioral habituation to the familiar stimulus orientation, suggesting shared local mechanisms of synaptic modification (Cooke et al., 2015; Kaplan et al., 2016). Thus, SRP may offer a direct readout of plasticity in the early visual system contributing to long-term recognition memory and habituation processes. Although changes in primary sensory activity to passive and reinforced visual stimulation have been extensively characterized (Aton et al., 2014; Kato et al., 2015; Makino and Komiyama, 2015; Poort et al., 2015; Kaneko et al., 2017; Gao et al., 2021), the sites and essential mechanisms that yield the observed response patterns remain poorly defined. In the case of SRP, it is still unclear how plasticity manifests outside of superficial cortical layers. This information is critical to pinpoint the primary site(s) of synaptic modification that store this tractable and foundational form of memory.

Several striking attributes of SRP had previously suggested the occurrence of thalamocortical (TC) Hebbian synaptic plasticity. First, long-term potentiation (LTP) induced in V1 by theta-burst electrical stimulation of the dorsal lateral geniculate nucleus (dLGN) both mimics and occludes SRP recorded in V1 L4 (Cooke and Bear, 2010). Second, both LTP and SRP are dependent on NMDA receptor (NMDAR) activity in V1 (Kirkwood and Bear, 1994; Collingridge, 2003; Frenkel et al., 2006; Cooke et al., 2015) and can be prevented by interfering with delivery of AMPARs to postsynaptic membranes (Frenkel et al., 2006). Third, SRP is eye-specific, indicating that modifications likely occur at TC synapses conveying information exclusively from one eye, before binocular integration by V1 neurons (Frenkel et al., 2006; Cooke et al., 2015). Hence, an early model proposed that long-term visual recognition memory is stored as NMDAR-dependent potentiation of TC synapses within V1 (Montgomery et al., 2021). Subsequent observations challenged this simple view, however. First, pharmacological elimination of intracortical activity that spares TC synaptic transmission in L4 abolishes SRP expression (Cooke and Bear, 2014; Kaplan et al., 2016). Second, the activity of specific populations of GABAergic inhibitory neurons in V1 is modified with visual experience and significantly diverges for familiar and novel stimuli (Hayden et al., 2021). Third, SRP expression in L4 VEPs is disrupted by selective perturbations of activity in cortical parvalbumin-expressing (PV+) interneurons (Kaplan et al., 2016). Fourth, selective knock-out (KO) of NMDARs from principal cells in L4, the primary target of TC input to V1, spares both SRP of L4 VEPs and behavioral habituation (Fong et al., 2020). Fifth, short-latency responses recorded in L4 at the onset of familiar and novel stimuli are indistinguishable, with differences emerging only over hundreds of milliseconds and not manifesting in VEPs until the second phase reversal (Hayden et al., 2021). Together, these findings suggest that SRP is not a direct readout of feedforward potentiation in L4. Instead, it is likely to involve intracortical plasticity and/or feedforward TC potentiation at still unidentified synapses outside of L4, which nevertheless influence responses in L4.

To advance our understanding of where and how synaptic plasticity in V1 serves visual recognition memory, in the current study we first investigated how manipulating NMDARs in neurons outside of L4 influences SRP measured with VEPs within L4, then went on to analyze SRP expression across all layers of V1. We confirmed that V1 glutamatergic neurons play a key role in SRP by using an intersectional genetic strategy to knock out NMDARs in only principal cells. We next targeted NMDARs in a genetically defined subset of excitatory neurons in L6 that are known to influence PV+ inhibition in L4 (Bortone et al., 2014; Guo et al., 2017), and found that the effect on SRP mimicked the pattern observed following NMDAR knock-out in excitatory neurons across all layers of V1. The discovery that a molecular manipulation in the deep layers disrupts SRP manifesting in L4 VEPs motivated us to then use high-density linear electrode arrays to simultaneously record neural activity across all layers of V1 after 6 d of SRP induction. The observed changes in both superficial and deep cortical layers are consistent with data previously obtained from L4 (Cooke et al., 2015; Hayden et al., 2021). Namely, phase reversals of familiar stimuli evoke larger amplitude VEPs across all layers compared with novel stimuli. Blocks of familiar stimuli also increase low frequency oscillatory power across all layers, whereas novel stimuli increase high frequency power except in the deepest portions of visual cortex. We confirm the previous finding that there is elevated peak L4 unit activity immediately following each phase reversal of the familiar stimulus (Cooke et al., 2015) and extend this observation to reveal that this increase in peak firing rate is also apparent in superficial, middle, and deep layers. After this transient increase, however, there is an extended period of suppressed firing between phase reversals of familiar stimuli that is not apparent for novel stimuli. Thus, while the phasic spiking response produced by familiar stimulus onset is greater than for novel stimuli, the overall firing rate is reduced, in agreement with dominant repetition suppression literature (Grill-Spector et al., 2006) and previous calcium imaging studies (Kato et al., 2015; Makino and Komiyama, 2015; T. Kim et al., 2020). Together, these sustained changes during familiar innocuous stimuli are consistent with a shift in cortical processing mode that is influenced by NMDARs on L6 principal cells.

## Materials and Methods

### Animal subjects

All procedures adhered to the guidelines of the National Institutes of Health and were approved by the Committee on Animal Care at MIT. Mice were housed in groups of two to five same-sex littermates after weaning at postnatal day (P)21–P23. They had access to food and water *ad libitum* and were maintained on a 12/12 h light/dark cycle. For acute laminar electrophysiological recordings, we used male and female C57BL/6J mice (The Jackson Laboratory) bred in the MIT animal colony. Careful study has shown no sex differences in SRP (Schecter et al., 2023).

For the cell-type specific knock-out experiment, we used either an intersectional viral strategy in genetically modified mice or mouse lines were crossed to restrict *Grin1* deletion to subpopulations of excitatory neurons. In both cases, mice expressing loxP sites around both copies of the *Grin1* gene were used to ablate the GluN1 subunit and, thereby, functional NMDARs in a Cre-dependent manner (*Grin1*^fl/fl^, The Jackson Laboratory, RRID:IMSR_JAX:005246; Tsien et al., 1996). For the local excitatory cell-specific GluN1 knock-out in V1, AAV8-CaMKIIa-Cre-GFP (knock-out group) or AAV8-CaMKIIa-GFP (control group; UNC Vector Core, 4.4 × 10^11^ vg/ml in sterile saline) was injected bilaterally into binocular V1 of *Grin1*^fl/fl^ animals (see surgical methods, below). To target the GluN1 knock-out to L6 cortico-thalamic neurons, *Grin1*^fl/fl^ mice were instead crossed with the previously described *Ntsr1-Cre* recombinase mouse line (B6.FVB(Cg)-Tg(Ntsr1-cre)GN220Gsat/Mmucd; *Ntsr1-Cre*, *Layer-6*, GENSAT, RRID:MMRRC_030648-UCD; Gong et al., 2007). Experimental subjects were *Grin1*^fl/fl^ and either Ntsr1-Cre^+/−^ (knock-out group) or Ntsr1-Cre^−/−^ (control group). To histologically validate Cre-expression, *Ntsr1*-Cre mice were also crossed with a Cre-reporter mouse line (B6.Cg-Gt(ROSA)26Sortm14(CAG-tdTomato)Hze/J; Ai14-tdTomato, The Jackson Laboratory, RRID:IMSR_JAX:007914).

### Surgery

**Figure 1.**
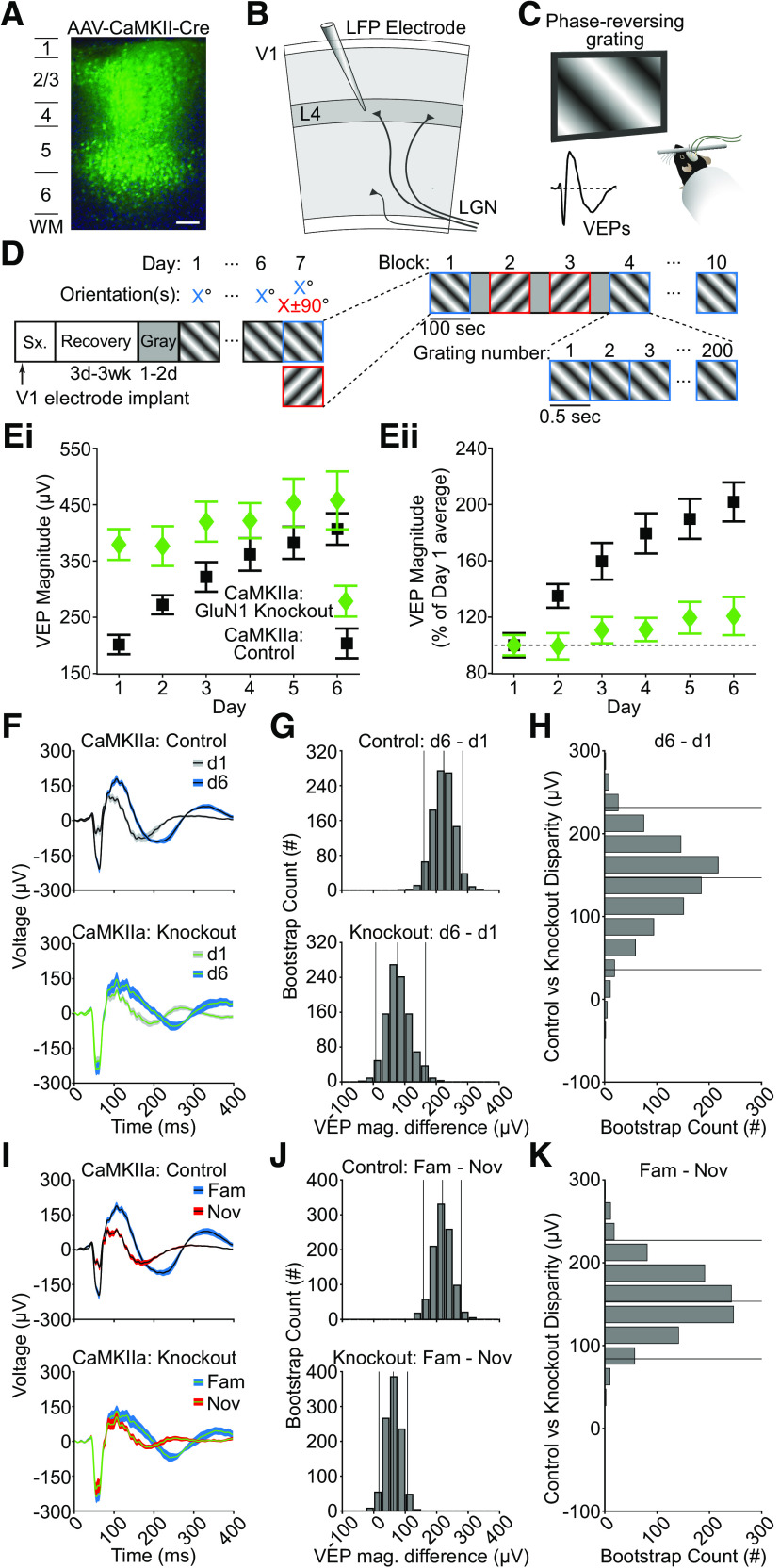
NMDA receptor knock-out in V1 excitatory neurons affects L4 VEPs and SRP. ***A***, A representative confocal micrograph of virally-induced fluorescent protein co-expression following injection of AAV8-CaMKIIa-Cre-GFP, a proxy for the spatial distribution of Cre-mediated NMDA-receptor knock-out across layers of V1 (green, GFP; blue, Hoechst counterstain; scale bar: 100 µm). ***B***, Diagram of visual cortex showing the electrode placement within L4. ***C***, Averaged VEPs elicited by phase reversals of full field, oriented, sinusoidal grating stimuli acquired binocularly from awake, head-fixed mice. ***D***, For 6 d, mice saw five blocks of phase-reversing gratings of a single orientation (100 phase reversals at 2 Hz), separated by 30 s of gray screen. On day 7, five blocks of the now familiar orientation were pseudo-randomly interleaved with five blocks of a novel stimulus (rotated 90° from the familiar orientation). ***Ei***, VEP magnitude plotted over days for both CaMKIIa-GluN1KO and control groups (symbol and bars represent mean ± SEM). ***Eii***, VEP magnitude as a percent of the group's average day 1 VEP magnitude plotted over days (symbol and bars represent mean ± SEM). ***F***, As a result of multiple days of experience, control animals had increased VEPs to the same stimulus on day 6 compared to day 1 (*n* = 13 mice). CaMKIIa-GluN1 KO animals did not show a similar increase (*n* = 14 mice). ***G***, The bootstrapped L4 VEP magnitude difference between day 6 and day 1 was large in control animals and small in KO animals. ***H***, The group disparity value is plotted, which compares bootstrapped day 6 – day 1 VEP magnitude differences between the control and knock-out mice. The disparity shows that the KO group undergoes less potentiation as a result of daily stimulus exposure. ***I–K***, Same as in ***F–H***, but comparing a novel stimulus on day 7 to the familiar stimulus on day 7. The control group has a larger VEP magnitude difference as a result of stimulus-dependent experience compared to knock-out animals. The left and right vertical lines in ***G*** and ***J*** are the 95% confidence intervals generated by the bootstrap procedure. The middle vertical line is the median bootstrapped difference. Similarly, in ***H*** and ***K***, the top and bottom vertical lines are the 95% confidence intervals for the disparity between the VEP magnitude differences of control and knock-out animals, whereas the middle line is the median bootstrapped difference.

**Figure 2.**
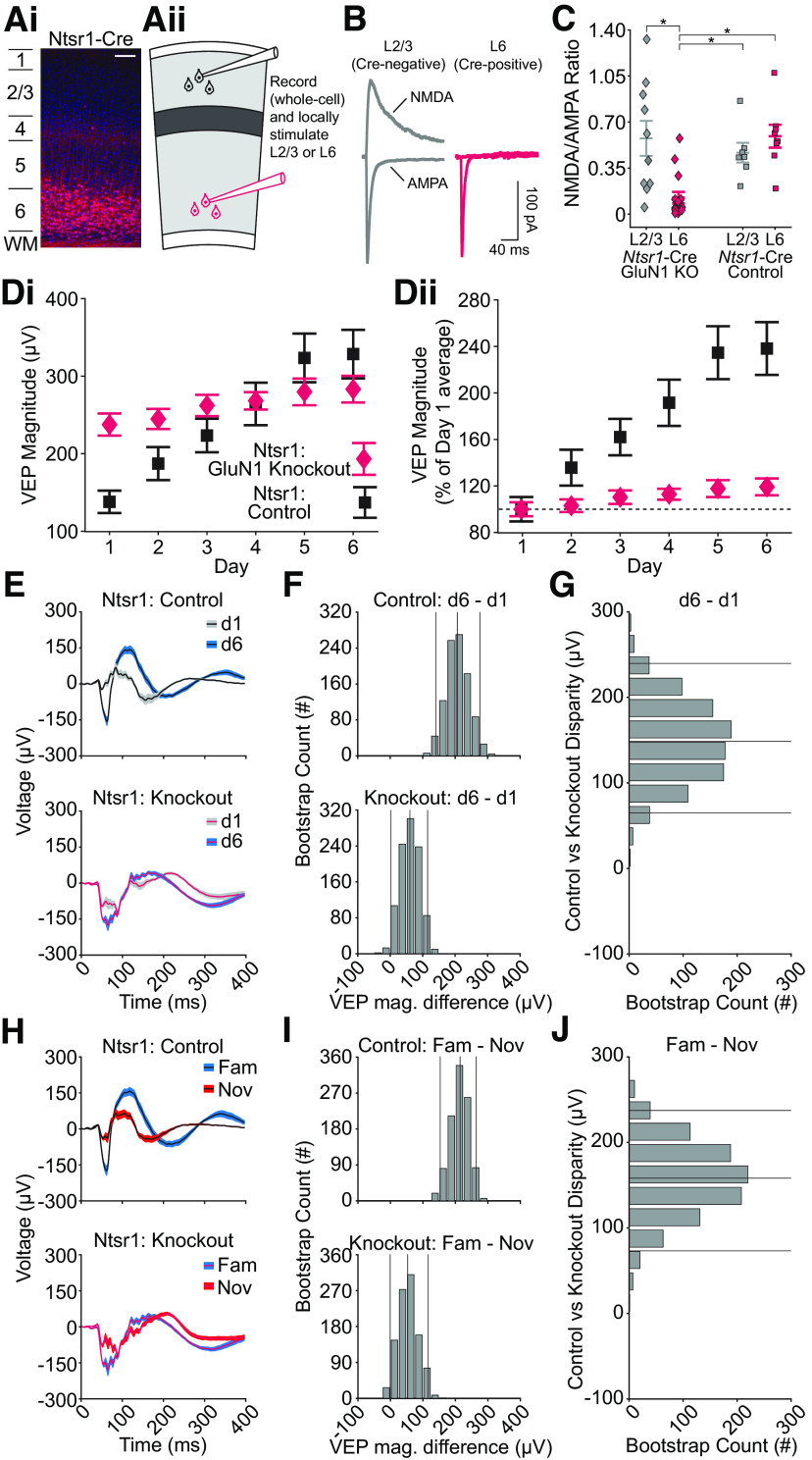
NMDA receptor knock-out in L6 excitatory neurons affects L4 VEPs and SRP. ***Ai***, Representative confocal micrograph of fluorescent Cre reporter tdTomato across layers of V1 in a Ntsr1-Cre^+/−^, Ai14^+/−^ mouse, illustrating the pattern of NMDA-receptor knock-out in Ntsr1-Cre^+/−^, Grin1^fl/fl^ mice (red, tdTomato; blue, Hoechst counterstain; scale bar: 100 µm). ***Aii***, Diagram of visual cortical slice showing electrode placement for L2/3 or L6 voltage clamp recordings during within-layer stimulation. ***B***, Sample traces of evoked NMDA and AMPA receptor currents from V1 principal cells in a L6-GluN1 knock-out animal (Ntsr1-Cre^+/−^, GluN1^fl/fl^) recorded from a fluorescent (Cre-positive, right) and a nonfluorescent (Cre-negative, left) cell. Scale bars: 40 ms, 100 pA. ***C***, Mean NMDA/AMPA receptor current ratio from cells in animals possessing two floxed copies of the GluN1 allele (*n* = 4 animals, 10 cells in L2/3, 15 cells in L6) or from animals possessing at least one wild-type copy of GluN1 (*n* = 4 animals, 7 cells in L2/3, 8 cells in L6). Horizontal bars represent mean ± SEM. An asterisk indicates the compared cells exhibited a significant difference in NMDAR/AMPAR current ratio, as determined by hierarchical bootstrapping. ***Di***, VEP magnitude plotted over days for both Ntsr1-GluN1 KO and control groups (symbol and bars represent mean ± SEM). ***Dii***, VEP magnitude as a percent of the group's average day 1 VEP magnitude plotted over days (symbol and bars represent mean ± SEM). ***E***, As a result of multiple days of experience, control animals had increased VEPs to the same stimulus on day 6 compared to day 1 (*n* = 12 mice). Ntsr1-GluN1 KO animals showed a smaller increase over days (*n* = 15 mice). ***F***, The bootstrapped L4 VEP magnitude difference between day 6 and day 1 was observed in both control and KO animals. ***G***, The group disparity value is plotted, which compares bootstrapped day 6 – day 1 VEP magnitude differences between the control and knock-out mice. The disparity shows that the L6 NMDA receptor knock-out group undergoes reduced VEP magnitude change as a result of stimulus experience. ***H–J***, Same as in ***E–G***, but comparing a novel stimulus on day 7 to the familiar stimulus on day 7. The knock-out group shows reduced levels of stimulus-dependent plasticity. The left and right vertical lines in ***F*** and ***I*** are the 95% confidence intervals generated by the bootstrap procedure. The middle vertical line is the median bootstrapped difference. Similarly, in ***G*** and ***J***, the top and bottom vertical lines are the 95% confidence intervals for the disparity between the VEP magnitude differences of control and knock-out animals, whereas the middle line is the median bootstrapped difference.

For layer 4 (L4) VEP recordings in cell-type specific knock-out experiments (Figs. 1, 2), young adult mice were injected subcutaneously with 0.1 mg/kg Buprenex and 1.0 mg/kg Meloxicam to provide analgesia. Induction of anesthesia was achieved via inhalation of isoflurane (3% in oxygen) or intraperitoneal injection of ketamine (50 mg/kg) and xylazine (10 mg/kg). Thereafter, anesthetic plane was maintained via inhalant isoflurane (∼1–2% in oxygen) for the duration of surgery (30–60 min). Before surgical incision, the head was shaved and the scalp cleaned with povidone–iodine (10% w/v) and ethanol (70% v/v). The scalp was resected along the midline and the skull surface was scored with a scalpel blade, and residual connective tissue removed with sterile saline and a cotton-tipped applicator. A steel headpost was affixed to the skull (anterior to bregma) with cyanoacrylate glue, which was used to position the mouse in a stereotaxic surgical frame (model 960 or 963, Kopf Instruments). Small burr holes were drilled above both hemispheres of binocular V1 (3.0–3.1 mm lateral of λ). For *Grin1*^fl/fl^ mice receiving AAV injections, a glass capillary tube (model 3-000-203-G, Drummond Scientific Company) was backfilled with mineral oil before being loaded into a Nanoject III injector (model 3-000-207, Drummond) affixed to the stereotaxic arm. The capillary was front-loaded with diluted AAV and gradually lowered ∼750 into V1, then retracted to 700 μm and permitted to equilibrate for 2–5 min before commencing injections. A total of six injection pulses of 13.8 nl were performed at each of three cortical depths (700, 450, and 250 μm), with 20 s between pulses and 120 s between depths. The pipette was slowly retracted 5–10 min after completion of the final injection, which reduces AAV backflow from the target site. For all NMDAR knock-out mice, tapered blunt-tip 300–500 kΩ tungsten recording electrodes (model 30070, FHC; 125-μm diameter shank) were implanted in each hemisphere, 450 μm below cortical surface. Silver wire (A-M Systems) reference electrodes were placed over right frontal cortex. Electrodes were secured using cyanoacrylate glue and the skull was covered with dental cement (Ortho-Jet, Lang Dental). Meloxicam (1 mg/kg) was administered daily on return to the home cage for 48–72 h following surgery. Signs of infection and discomfort were carefully monitored. Mice were allowed to recover for at least 48 h before head-fixation.

For laminar recordings, young adult C57BL/6J mice (*n* = 8 female, 10 male) were first injected with 5.0 mg/kg Meloxicam subcutaneously to provide analgesia. Induction of anesthesia was achieved via inhalation of isoflurane (3% in oxygen) and thereafter maintained via inhalant isoflurane (∼1–2% in oxygen). Before surgical incision, the head was shaved and the scalp cleaned with povidone–iodine (10% w/v) and ethanol (70% v/v). Scalp was resected and the skull surface was scored. Then, a stainless-steel head plate was attached to the skull and a 3-mm craniotomy was made over binocular V1. A sterile 3-mm round glass coverslip (CS-3R-0, Warner Instruments) was gently laid on top of the exposed dura mater and held in place with VetBond (3M) and thin Ortho-Jet bridges (Lang Dental). A silver wire (A-M Systems) reference electrode was inserted into left frontal cortex. Dental acrylic (C&B Metabond Quick Adhesive Cement System) was mixed and applied throughout the exposed skull surface. The coverslip was covered with Kwik-Sil (World Precision Instruments). Slow-releasing Buprenorphine was administered on return to the home cage (1 mg/kg, s.c.). Signs of infection and discomfort were carefully monitored. A total of seven AAV-injected mice were excluded from analysis because of poor recording quality, aberrant viral transduction/recombination, and/or health/technical issues.

On the morning of the acute recording, mice were once again isoflurane anesthetized and injected with 0.1 mg/kg Buprenex subcutaneously to provide analgesia. The Kwik-Sil was removed, the Ortho-Jet bridges broken, and the coverslip carefully pried off with a hooked needle. The craniotomy and surrounding area were filled with HEPES + agar. This mixture consists of 0.2 × *g* high EEO Agarose (Sigma-Aldrich), 0.21 g Certified Low-Melt Agarose (Bio-Rad), and 10.25 ml artificial CSF (ACSF) + HEPES (150.0 mm NaCl, 2.5 mm KCl, 10.0 mm HEPES, 2.0 mm CaCl_2_, and 1.0 mm MgCl_2_). Kwik-Sil was applied again to prevent the agar from drying. This process was typically completed within 1 h. Nonsteroidal anti-inflammatory drugs were administered on return to the home cage (meloxicam, 1 mg/kg, s.c.). The mice were head fixed, the Kwik-Cast removed, and a 64-channel laminar probe (Cambridge NeuroTech) was inserted slowly (∼100 μm/min) into V1 perpendicular to the cortical surface. Recordings were obtained and the mouse was euthanized immediately thereafter. We eliminated six mice because of either poor electrode placement (as measured by histology or atypical VEPs, *n* = 3), user error in recording (*n* = 1), superficial damage (*n* = 1), or surgical loss (*n* = 1). The remaining animals (*n* = 12; 6 female, 6 male) were included in all laminar analyses.

### Acute slice whole-cell electrophysiology

Functional loss of NMDAR expression in *Ntsr1-Cre* x *Grin1*^fl/fl^ animals was confirmed via *ex vivo* electrophysiology in slices obtained from dedicated cohorts of experimentally naive mice. Fluorescence-guided whole-cell voltage clamp recordings were used to measure AMPA and NMDA receptor mediated EPSCs in Cre positive-cells from knock-out mice (Cre^+/−^, *Grin1*^fl/fl^) and age-matched control animals hemizygous for Cre but with at least one wild-type copy of *Grin1* (i.e., Cre^+/−^, *Grin1*^fl/+^ or Cre^+/−^, *Grin1*^+/+^). Two strategies were used to fluorescently label cells with Cre recombinase activity. First, we injected an adeno-associated virus (AAV5-EF1α-DIO-GFP, UNC viral core) into the binocular zone of V1 to drive Cre-mediated expression of the green fluorescent protein (GFP) reporter (3.1 mm lateral of λ, 81 nl of virus at each of 3 depths: 600, 450, and 300 μm from the cortical surface), allowing three to four weeks of recovery before tissue harvest. Surgical injections were performed using a Nanoject II system (model 3-000-204, Drummond Scientific) as described above, except the mice were positioned in the stereotaxic frame using earbars. Second, we bred a triple transgenic animal using the Cre-driver x *Grin1^f^*^l/fl^ lines crossed with the Cre-dependent tdTomato reporter line, Ai14. In both cases, animals were approximately six months old at time of slice preparation. Coronal slices of V1 were prepared at a thickness of 350 μm in ice-cold dissection buffer containing (in mm): 87 NaCl, 75 sucrose, 2.5 KCl, 1.25 NaH_2_PO_4_, 25 NaHCO_3_, 0.5 CaCl_2_, 7 MgSO_4_, 1.3 ascorbic acid, and 10 D-glucose, saturated with 95% O_2_ and 5% CO_2_. Slices were recovered for 40 min at 33°C and for ∼1 h at room temperature in artificial CSF (ACSF) containing (in mm): 124 NaCl, 5 KCl, 1.23 NaH_2_PO_4_, 26 NaHCO_3_, 2 CaCl_2_, 2 MgCl_2_, and 10 D-glucose, saturated with 95% O_2_ and 5% CO_2_. Whole-cell patch clamp recordings were performed in continuous perfusion of carbogenated artificial CSF (ACSF) at 30°C using borosilicate pipettes with tip resistances of 3–5 MΩ. Pipettes were filled with balanced intracellular solutions containing (in mm): 115 cesium methane-sulfonate (CsMeSO_3_), 2.8 NaCl, 0.4 EGTA, four ATP-Mg^2+^, 10 Na^+^-phosphocreatine, 0.5 Na^+^-GTP, 5 TEA-Cl-, 5 QX-314 Br- buffered with 20 HEPES (pH 7.25, 290 mOsm). Layer 6 and layer 2/3 EPSCs were evoked by stimulation of layer 6 and layer 2/3, respectively, in the *Ntrs1*-Cre line (150 μs, 0.1 Hz, glass pipette electrode, and WPI A365 stimulus isolator) at holding potentials of −70 and +40 mV. The AMPA receptor component was measured from evoked EPSCs at −70 mV in the presence of picrotoxin (100 μm) and glycine (1 μm), and the NMDA receptor component was measured from evoked EPSCs at +40 mV in the presence of DNQX (20 μm). NMDA/AMPA receptor mediated EPSC ratios were calculated on a cell-by-cell basis and hierarchical bootstrapping was used to evaluate differences between groups.

### Visual stimulus delivery

For the cell-specific knock-out mouse lines, after recovery from electrode implantation, experimentally naive mice were acclimated to head restraint in front of a gray screen for a 30-min session on each of 2 consecutive days. After this acclimation period, mice received 6 d of passive exposure to an oriented grating stimulus at a set, noncardinal orientation. Each session began with 5 min of gray screen, after which they were presented with 5 blocks of 100 phase-reversals of an oriented grating stimulus, phase-reversing at 2 Hz. A 30-s period of gray screen was presented between each stimulus block. On day (d)7, mice were shown blocks of the familiar stimulus orientation pseudorandomly interleaved with blocks of a novel stimulus offset by ±90°.

For the laminar recordings, after recovery from the headplate surgery, mice were acclimated to head restraint in front of a gray screen for a 60-min session on each of 2 consecutive days. After habituation, mice were presented with 10 blocks of 100 phase-reversals of an oriented grating stimulus phase-reversing at 1 Hz. They were shown this stimulus for 4 consecutive days. On day 5, they were shown five blocks of the familiar stimulus orientation pseudo-randomly interleaved with five blocks of a novel stimulus offset 90° from the familiar orientation. Each stimulus block was preceded by a period of gray screen, a period of black screen, and another period of gray screen. Gray periods and black periods lasted 10 s each, for a total of 30 s of preblock activity. Discrete sections of gray and black screen viewing were timestamped for later normalization.

Visual stimuli consisted of full-field, 0.5 cycles/°, 100% contrast, sinusoidal gratings that were presented on a computer monitor. Visual stimuli were generated using custom software written in either C++ for interaction with a VSG2/2 card (Cambridge Research Systems) or MATLAB (MathWorks) using the PsychToolbox extension (http://psychtoolbox.org) to control stimulus drawing and timing (https://github.com/jeffgavornik/VEPStimulusSuite). Grating stimuli spanned the full range of monitor display values between black and white, with γ-correction to ensure gray-screen and patterned stimulus conditions are isoluminant.

### *In vivo* electrophysiology experimental design and analysis

Electrophysiological recordings were conducted in awake, head-restrained mice. Recordings were amplified and digitized using the Recorder-64 system (Plexon Inc.) or the RHD Recording system (Intan Technologies). For the cell-specific knock-out mice, two recording channels were dedicated to recording continuous local field potential (LFP) from V1 in each implanted hemisphere. Local field potential was recorded from V1 with 1-kHz sampling using a 500-Hz low-pass filter. For the laminar recordings on the Intan system, we sampled at 25 kHz and used a 0.1-Hz high-pass and a 7.5-kHz low-pass filter. Local field potential data were imported (see Importing and data cleaning) and the local field potential's spectral content was analyzed (see below, Spectral analysis).

### Data import and cleaning

All analyses were conducted using custom MATLAB code and the Chronux toolbox (Bokil et al., 2010). For cell-specific knock-out mice, the local field potential data were imported and converted to microvolts (µV). A handful of these recordings had minor errors in the event codes that were corrected *post hoc*. For laminar recordings, the raw 25-kHz data from each channel were extracted and converted to µV. Then they were downsampled to 1000 Hz and a third order 1- to 300-Hz Butterworth filter was applied. For all data, the mean of the entire channel's data was subtracted from each time point to account for any DC offset in the system. Next, the data were locally detrended using the locdetrend function of the Chronux toolbox using a 0.5 s window sliding in chunks of 0.1 s. Finally, a third-order Butterworth filter was used to notch frequencies between 58 and 62 Hz. For the multiunit activity of laminar recordings, the raw 25-kHz data were extracted for each channel. A 60 Hz, 10-dB bandwidth IIR notch filter was applied to each channel and the median value of each channel was subtracted from said entire channel. Finally, the median value across channels for each time point was subtracted from all channel's timepoints. These data were stored separately for later use (see below, Multiunit activity analysis). Visually evoked potentials were normalized by subtracting the average of the first 10 ms of each trial from that trial. A 10-ms moving Gaussian was applied to smooth the visually evoked potential waveform.

### Current-source density analysis and laminar identification

Performing a current-source density (CSD) analysis on laminar local field potential (LFP) data in V1 allowed us to identify layer 4 (L4) and align our data (Mitzdorf, 1985, 1987; Aizenman et al., 1996). The LFP data were temporally smoothed with a 20-ms Gaussian window. We then used a five-point hamming window to compute the CSD using the standard formula (Ulbert et al., 2001; Speed et al., 2019). We refer to L4 as 0 µm, the site of the earliest and deepest sink immediately below the superficial source. All other channels were referenced according to that landmark. Thus, superficial layers were the channels above 0 µm and deep layers were the channels below 0 µm. For the sake of reporting in Results, we have broken the laminar data into four segments: L2/3 is above +90 µm, L4 is between +90 and −70 µm, L5 is between −70 and −210 µm, and L6 is below −210 µm. Reported statistics look for the largest significant difference within those four bounds.

### Spectral analysis

Given that the visually evoked potential violates assumptions required for spectral analysis (namely, second-order stationarity), we only analyzed the spectral activity between 400 and 1000 ms after a phase-reversal. We computed the multitapered spectrum of the local field potential using the Chronux toolbox (Bokil et al., 2010). We used zero-padding to the second power, five tapers, and a time-bandwidth product of 3. To calculate the normalized spectrum/spectrogram, we found the median spectrum/spectrogram of the animal's black screen and took 10*log10(stimulus_spectrum/median_black_spectrum). This is reported as a decibel (dB).

### Multiunit activity analysis

To obtain the multiunit activity, we calculated a value known as multiunit activity envelope (MUAe). MUAe provides an instantaneous measure of the number and size of action potentials of neurons in the vicinity of the electrode tip (Legatt et al., 1980; Brosch et al., 1995; Super and Roelfsema, 2005). It does not depend on the arbitrary positioning of a threshold level and therefore does not select only large spikes. To obtain this value, a third order bandpass (500–5000 Hz) Butterworth filter was applied to the common median referenced data that was previously stored (see Importing and data cleaning). This step eliminates low-frequency field potentials and isolates spiking unit activity. Next, the absolute values of the data were taken (units of µV) to help remove contamination from far-field signals. Finally, a third order low pass (<250 Hz) Butterworth filter was applied to smooth the signal and the data were downsampled to 1000 Hz to align with local field potential data.

For z-scored data, the average across all trials for a given stimulus was calculated. Then, for each channel and animal, the average and SD of the familiar and novel MUAe postphase-reversal (up to 400 ms) was found. This was done by creating two [nSamplesWithinTrials × nDepths × nAnimals] matrices for familiar and novel stimuli where each index corresponds to the average value across all trials of said sample, depth, and animal. The SD for the familiar and novel matrices were taken with respect to the first dimension (i.e., the SD of nSamplesWithinTrials). This created two matrices of [1 × nDepths × nAnimals] that represent the SD value for a given depth and animal. The familiar and novel matrices are averaged together to get an average SD value for each depth and animal. A similar method computed the mean value. These two [1 × nDepths × nAnimals] matrices for the average mean and the average SD were then used with the familiar and novel [nSamplesWithinTrials × nDepths × nAnimals] matrices to calculate the *z* score via matrix algebra. Note that both the familiar and novel mean and SD were averaged together such that both familiar and novel stimuli could be z-scored to the same values (thus allowing direct comparison).

### Disparity

To compare experience-dependent changes between two groups we create a term we call “disparity.” This value represents the difference of a difference and is created via nonparametric hierarchical bootstrapping (see below, Statistics). Briefly, for each group, a random selection of animals (with replacement) was selected. Each animal saw both stimuli, so a random selection of trials (with replacement) was further selected. The difference between the stimulus trials was calculated and averaged within a group, resulting in an average (randomly selected) stimulus difference for each group. The difference of these stimulus difference values, the “disparity,” was calculated and stored. The process was repeated 1000 times yielding a distribution that would include 0 if the two groups had similar stimulus differences.

### Statistics

All statistics were done with the nonparametric hierarchical bootstrap for multilevel data (Saravanan et al., 2020). Briefly, statistical comparisons were between two groups, designated A and B. Most of our experiments used a within-animal design wherein animals experienced both familiar and novel stimuli (or stimuli on day 6 and day 1). In these cases, each animal in the experiment could and did contribute to both Group A and Group B. However, when comparing one genotype to another, each mouse could only contribute to the one appropriate genotypic group. To begin the bootstrap process, mice were randomly selected with replacement from the experimental population. For within-animal design comparisons, a random selection (with replacement) of mice was chosen and for each randomly chosen mouse a random selection (with replacement) of both Group A trials and Group B trials was selected. For genotype comparisons, a random selection (with replacement) of mice was chosen from each genotype and for each randomly chosen mouse a random selection (with replacement) of trials was selected to go into either Group A or Group B based on the genotype. Once all data were randomly selected, a statistic (e.g., VEP magnitude, spectral power, multiunit activity, etc) was computed from the trial and the mean difference between group A and group B was stored. This entire bootstrap process was repeated 1000 times. Once all 1000 bootstraps had been completed, the bootstrapped differences were sorted from lowest to highest value. The 500th value was the median group difference and is plotted in most graphs. The 25th value was the lower bound of the 95% confidence interval and the 975th value was the upper bound of the 95% confidence interval. Since the acute laminar recording experiments made multiple comparisons, we used the more conservative 99% confidence interval (CI) when making inferences. These used the fifth and 995th values of the sorted bootstrap. If the 95% or 99% confidence interval did not include zero, we report a statistically significant difference between Group A and Group B. This is reported as either an asterisk near the corresponding data on the plot or as a separate three-color plot with the valence and significance corresponding to a given color.

## Results

### Deletion of NMDARs in V1 principal cells disrupts SRP

In canonical models of the neocortical microcircuit, excitatory cells in layers 4 and 6 receive the bulk of feedforward projections from sensory thalamus (Douglas and Martin, 2004). Yet, surprisingly, selective knock-out of NMDARs from the excitatory cells in L4 has no impact on SRP (Fong et al., 2020). Given the critical involvement of PV+ cells in SRP expression (Kaplan et al., 2016), the differential modulation of distinct V1 interneuron populations by familiar and novel stimuli (Hayden et al., 2021), and the fact that PV+ neurons receive excitatory input from the thalamus (Cruikshank et al., 2007), we first sought to confirm whether SRP relies on NMDARs on excitatory cells at all.

To do so, we knocked out the obligatory GluN1 subunit of the NMDAR in excitatory neurons of V1 by taking advantage of their selective expression of α Calcium/Calmodulin-dependent Kinase II (CaMKIIa). Recombination-mediated excision of the *Grin1* gene was targeted to excitatory cells across all cortical layers by intracranial microinjection of AAV8-CaMKIIa-Cre-GFP into transgenic mice in which Cre-dependent loxP sites were inserted around both copies of the *Grin1* gene (*Grin1*^fl/fl^). Thus, NMDARs were functionally ablated in only V1 excitatory neurons, as illustrated by histologic examination of GFP expression induced by the AAV (Fig. 1*A*). Control mice were infused with AAV8-CaMKIIa-GFP to produce equivalent fluorophore expression in the same excitatory cell population without Cre-mediated ablation of NMDARs. During the same surgery, local field potential recording electrodes were implanted within layer four and headposts affixed to the skull surface of all mice for subsequent acquisition of local field potential (LFP) data from V1 (Fig. 1*B*). Following three to four weeks of surgical recovery and 2-d acclimation to the experimental apparatus, awake, head-fixed mice viewed 5 min of gray screen, before the onset of isoluminant full field, 2-Hz phase-reversing sinusoidal grating stimuli separated into 5 blocks of 100 phase-reversal pairs, with each block separated by 30 s of gray screen (Fig. 1*C*,*D*). Aligning and averaging all stimulus-evoked LFP waveforms occurring within a 400-ms time window from the start of each phase-reversal revealed a stereotyped visually evoked potential (VEP), with an average magnitude that increased across days of exposure in controls, as expected (Fig. 1*E*). However, on day 1, the trough-to-peak magnitude of VEPs acquired from control *Grin1*^fl/fl^ mice that had received AAV8-CaMKIIa-GFP virus was significantly lower than for their *Grin1*^fl/fl^ littermates that had received AAV8-CaMKIIa-Cre-GFP to knock-out (KO) NMDAR expression in excitatory neurons (Fig. 1*F*, median trough-to-peak VEP magnitude difference: −146.81 µV, 95% CI = [−221.67 −80.53] µV, *n* = 13 Cre^−^ control mice, 14 Cre^+^ KO mice). Thus, baseline differences in response magnitude must be considered when comparing data from the control and KO groups.

We next sought to determine how postnatal deletion of functional NMDARs in V1 excitatory cells influences SRP. We induced SRP using a standard protocol, exposing mice to a phase-reversing stimulus at the same orientation each day for 6 consecutive days. We used nonparametric hierarchical bootstrapping for both visualization and statistical evaluation of the data. Briefly, we conducted random draws (with replacement) from the animals and trials that comprised a group, calculated a statistic (such as VEP magnitude) from the average data, then repeated this process 1000 times to generate a distribution. Histograms from this bootstrap were created to aid visualization of the statistical comparison being made (Fig. 1*G*). Consistent with previous studies (Frenkel et al., 2006; Cooke et al., 2015; Kaplan et al., 2016; Fong et al., 2020; Hayden et al., 2021), VEP magnitudes increased over days in control animals (Fig. 1*F*,*G*, top panel) such that the VEP on day 6 was significantly larger than d1 (Fig. 1*G*, top panel, median trough-to-peak VEP magnitude difference: 222.31 µV, 95% CI = [159.46 282.45] µV, *n* = 13 Cre^−^ control mice). For the KO mice, the magnitude also increased over days (Fig. 1*F*,*G*, bottom panel) and the VEP on d6 was significantly larger than d1 (Fig. 1*G*, bottom panel, median trough-to-peak VEP magnitude difference: 76.16 µV, 95% CI = [7.28 164.58] µV, *n* = 14 Cre^+^ KO mice), but the difference was clearly smaller than control animals.

To directly compare experience-dependent changes in the VEP magnitude between KO and littermate controls, we calculated a term we call the “disparity.” Again, using nonparametric hierarchical bootstrapping we randomly sampled and calculated the d6–d1 VEP magnitude difference for control animals and for KO animals. These two values were then compared, and the process was repeated (1000 times), yielding a distribution of disparities. If two groups expressed the same level of experience-dependent response plasticity, then the disparity would be 0 µV. However, if the control group expressed more experience-dependent response plasticity, then the disparity would be shifted toward positive numbers.

As expected from visual inspection of Figure 1*G*, CaMKIIa-GluN1 KO animals had significantly less experience-dependent response plasticity than their littermate controls (Fig. 1*H*, median disparity: 146.61 µV, 95% CI = [35.69 231.58] µV, *n* = 13 Cre^−^ control mice, 14 Cre^+^ KO mice). Combined with the day 1 data, this finding suggests that CaMKIIa-GluN1 KO animals either underwent reduced novelty-induced modulation on initial exposure to a grating stimulus or less experience-dependent potentiation with stimulus familiarization. Regardless, the effects of experience-dependent plasticity are clearly muted in these KO animals.

A disparity was also observed on day 7, when interleaved blocks of familiar and novel stimuli were shown. Control animals exhibited lower magnitude VEPs in response to novel stimuli (Fig. 1*I*, top panel), showing a statistically significant difference between the magnitude of VEPs produced by familiar and novel stimuli (Fig. 1*J*, top panel, median trough-to-peak VEP magnitude difference: 216.68 µV, 95% CI = [156.83 275.68] µV, *n* = 13 Cre^−^ control mice). For the KO mice, while a significant difference was apparent between familiar and novel VEPs (Fig. 1*I*, bottom panel), that difference was small (Fig. 1*J*, bottom panel, median trough-to-peak VEP magnitude difference: 61.43 µV, 95% CI = [16.02 106.15] µV, *n* = 14 Cre^+^ KO mice). A group difference in experience-dependent plasticity is inferred from the disparity between CaMKIIa-GluN1 KO animals and their littermate controls (Fig. 1*K*, median disparity: 153.46 µV, 95% CI = [84.01 227.00] µV, *n* =13 Cre^−^ control mice, 14 Cre^+^ KO mice). Thus, NMDARs in CaMKIIa-expressing excitatory principal neurons for required for the full induction and/or expression of SRP.

### Deletion of NMDAR in L6 cortico-thalamic neurons disrupts SRP

NMDAR expressed in excitatory neurons within L4 are not necessary for full SRP expression (Fong et al., 2020). Considered with the above data, we conclude that excitatory cells in other layers must be responsible for the NMDAR-dependence of SRP. Thus, we ablated NMDAR from a population of neurons in another thalamo-recipient layer, L6 (Fig. 2*Ai*), and tested for SRP deficits measured via the L4 VEP. We used a genetic strategy to create a L6 NMDAR knock-out mouse line by crossing the *Grin1*^fl/fl^ mice with Neurotensin Receptor 1 (Ntsr1)-Cre mice (see Materials and Methods), which express Cre within a population of excitatory neurons in L6 that project back to thalamus and have a characteristic pattern of intracortical connectivity (Gong et al., 2007). We confirmed that these mice lack NMDAR in L6 by whole-cell recording from pyramidal excitatory neurons (Fig. 2*Aii*). GFP-expressing L6 pyramidal neurons from Ntsr1-GluN1 KO mice had a significantly reduced NMDA/AMPA receptor ratio compared with control cells (Fig. 2*B*,*C*, all bootstrapped 99% confidence intervals between control groups and L6 KO cells did not include 0), indicating successful reduction in functional NMDARs. The VEP magnitude increased over days of exposure for both control and Ntsr1-Cre GluN1 KO mice, albeit control mice showed a greater increase relative to the day 1 response (Fig. 2*D*).

As with the CaMKIIa-GluN1 KO animals, on day 1 the VEP magnitude for the Ntsr1-GluN1 KO mice was increased compared with Cre^−/−^ littermate controls (Fig. 2*E*, median trough-to-peak VEP magnitude difference: −77.80 µV, 95% CI = [−144.19 −18.17] µV, *n* = 12 control mice, 15 KO mice). Thus, again, a baseline offset must be considered when comparing data from the Ntsr1-GluN1 KO and the control population. We then induced SRP over 6 d and found that the VEP of both KO and control groups increased over days (Fig. 2*E*,*F*) but, in another phenocopy of the CaMKIIa-GluN1 KO mice, the Ntsr1-GluN1 KO underwent notably less potentiation from day 1 onwards. For the littermate control group, the VEP magnitude on day 6 was significantly greater than day 1 (Fig. 2*F*, top panel, median trough-to-peak VEP magnitude difference: 206.79 µV, 95% CI = [140.89 276.15] µV, *n* = 12 control mice). For the KO mice, there was a smaller but still significant VEP difference between day 6 and day 1 (Fig. 2*F*, bottom panel, median trough-to-peak VEP magnitude difference: 60.61 µV, 95% CI = [2.27 115.15] µV, *n* = 15 KO mice). As expected from visual inspection of Figure 2*F*, Ntsr1-GluN1 KO animals have less experience-dependent plasticity than their littermate controls (Fig. 2*G*, median disparity: 148.59 µV, 95% CI = [65.06 239.78] µV, *n* = 12 control mice, 15 KO mice). Combined with the day 1 data (Fig. 2*E*) and like CaMKIIa-GluN1 KO animals, this finding suggests that Ntsr1-GluN1 KO animals either fail to potentiate with stimulus familiarity or fail to suppress cortical response when presented with a novel stimulus on day 1.

As expected, this group disparity is also seen in the familiar/novel difference (Fig. 2*H*). Control animals have lower magnitude VEPs in response to novel stimuli (Fig. 2*I*, top panel, median trough-to-peak VEP magnitude difference: 214.55 µV, 95% CI = [153.54 264.31] µV, *n* = 12 control mice). For the KO mice, while there was a slight difference between VEPs produced by familiar and novel stimuli (Fig. 2*H*), that difference was not significant (Fig. 2*I*, bottom panel, 95% CI includes 0, median trough-to-peak VEP magnitude difference: 53.20 µV, 95% CI = [−0.63 115.29] µV, *n* = 15 KO mice). This reduction in experience-dependent plasticity is revealed by the disparity between Ntsr1-GluN1 KO animals and their littermate controls (Fig. 2*J*, median disparity: 158.17 µV, 95% CI = [73.23 237.33] µV, *n* = 12 control mice, 15 KO mice). Thus, NMDARs are required in Ntsr1-expressing L6 principal cells for the full expression of SRP.

In addition to differences in absolute magnitude of VEPs in Ntsr1-GluN1 KO mice, we note that the morphology of the VEP looks different in comparison to the littermate controls, suggesting potential differences in the individual components of the VEP and latencies to these components. With this in mind, we calculated the negativity and latency to the N2 component. For the Ntsr1-GluN1 KO experiment, the disparity measure for N2 negativity suggested a slight difference between genotypes, but only when comparing day 6 to day 1 (median disparity: 51.49 µV, 95% CI = [0.66 100.47] µV, *n* = 12 control mice, 15 KO mice). However, there was a clear change in the latency when comparing day 6 to day 1 (median disparity: 69 ms, 95% CI = [22 119] ms, *n* = 12 control mice, 15 KO mice) and when comparing familiar to novel on day 7 (median disparity: 73 ms, 95% CI = [11 141] ms, *n* = 12 control mice, 15 KO mice). Interestingly, for the CaMKIIa-GluN1 KO experiment, the disparity measure for both N2 negativity and N2 latency is essentially centered on 0 (data not shown), suggesting GluN1 KO in all layers of V1 does not affect the expression of SRP in the later components. This is one way in which the Ntsr1-GluN1 KO does not phenocopy the GluN1 KO across all excitatory neurons in V1.

### Experience-dependent changes in the VEP and CSD are seen at multiple depths

**Figure 3.**
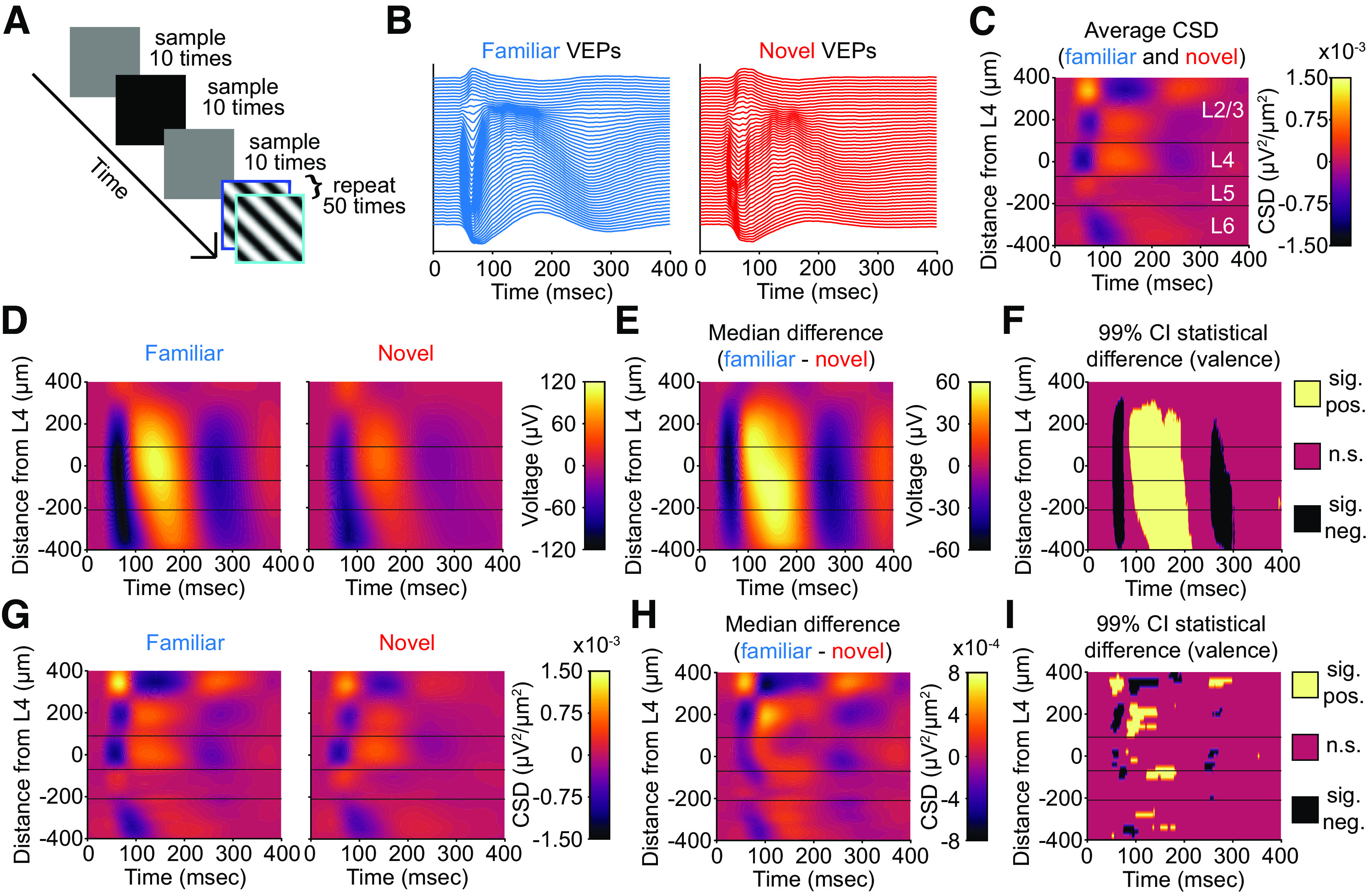
SRP of VEPs across multiple layers of V1. ***A***, We recorded extracellular activity from primary visual cortex (V1) in awake, head-fixed mice in response to phase-reversing sinusoidal grating stimuli (*n* = 12 mice). The experimental paradigm shows both gray and black screen stimuli between blocks of phase-reversing gratings, with the former ensuring isoluminance at the onset of high-contrast stimuli and the latter used for spectral normalization (see Materials and Methods). Cortical responses elicited by both the 0° (“flip,” blue) and 180° (“flop,” cyan) phases of stimulation are combined in subsequent analyses. ***B***, Laminar probes were inserted perpendicular to the cortical surface of binocular V1 and recordings made at 25 kHz. Plots display the low-pass local field potential from each site of the linear electrode array distributed along the *y*-axis according to laminar depth. Aligning to phase-reversal onset reveals stereotyped evoked potentials for familiar and novel stimuli. ***C***, The average current-source density plot for all phase-reversals for familiar and novel stimuli reveals L4 as the site of the earliest and deepest sink. ***D***, The average VEPs across all layers during familiar and novel stimulus blocks. ***E***, Nonparametric hierarchical bootstrapping results for median VEP differences across layers. ***F***, Regions of the laminar VEP in ***E*** where the bootstrapped 99% confidence interval does not include 0 (thus the difference is statistically significant). ***G–I***, As in ***D–F***, but for the current-source density analysis of the VEPs. All plots use an average smoothing kernel that spans 10% of each axis. Three horizontal lines arranged from top to bottom represent the approximate boundaries between L2/3, L4, L5, and L6.

To date, measurement of SRP has been restricted to L4 VEPs. The current finding that SRP is disrupted by deletion of NMDAR in L6 principal neurons highlights the paucity of data available for this form of experience-dependent plasticity in layers outside L4. Thus, we obtained acute, laminar recordings of V1 from awake, head-fixed, wild-type C57BL/6J mice to measure SRP across all layers simultaneously. After 2 consecutive days of habituation, mice were presented with 10 blocks of 100 1-Hz phase-reversals of an oriented grating stimulus. Preceding each block was a period of gray screen, then black, then gray (Fig. 3*A*). The gray screens were isoluminant with the phase-reversing grating to minimize pupil dilation artifacts at the onset and offset of stimulus blocks. The black screen was shown for spectral normalization because gray screens elicit luminance-dependent narrow-band oscillations at 60 Hz that are measured in the cortex but arise from subcortical sources (Saleem et al., 2017). This viewing protocol was shown for 4 consecutive days, and on day 5, we recorded acutely from V1 (see Materials and Methods for recording timeline) while presenting blocks of both familiar and novel stimuli in a pseudo-randomly interleaved manner.

On the recording day, we obtained VEPs across all layers for both familiar and novel stimuli (Fig. 3*B*). Performing current-source density (CSD) analysis on the VEPs across all layers allowed us to confirm the identity of L4, which is the earliest and deepest sink below the superficial source (Fig. 3*C*; Mitzdorf, 1985, 1987; Aizenman et al., 1996). Characteristic VEP morphology observed at this depth further confirmed the location. Visual inspection of the CSD suggested layer boundaries denoted by horizontal lines on laminar plots. These separate the data visually into L2/3, L4, L5, and L6, with statistics reported referencing to these laminar boundaries.

Previous work studying SRP has revealed that long-term stimulus familiarity increases the VEP magnitude in L4, increases the low-frequency (∼15 Hz) power, and decreases the high-frequency (∼65 Hz) power of the LFP recorded within L4 (Frenkel et al., 2006; Cooke and Bear, 2010; Cooke et al., 2015; Kaplan et al., 2016; Fong et al., 2020; T. Kim et al., 2020; Hayden et al., 2021). We wondered to what extent these changes are exclusive to L4. Plotting the VEP for both familiar and novel stimuli, as well as the difference between them, revealed that visual experience changes the VEP magnitude across most cortical laminae (Fig. 3*D–F*). In agreement with previous results, L4 shows a significantly different VEP peak-negativity to peak-positivity difference between familiar and novel stimuli (Fig. 3*E*,*F*; Table 1, left; 99% CI = [96.74 214.86] µV). This difference is also detectable in L2/3 (Fig. 3*E*,*F*; Table 1, left; 99% CI = [88.22 208.29] µV), in L5 (Fig. 3*E*,*F*; Table 1, left; 99% CI = [96.48 205.23] µV), and in L6 (Fig. 3*E*,*F*; Table 1, left; 99% CI = [74.24 170.58] µV). Thus, experience-dependent plasticity can be detected in superficial, middle, and deep layers of V1 using the VEP.

**Table 1.** Familiarity changes the VEP magnitude and CSD sink magnitude in different layers of V1

	VEP difference (F – N)			CSD sink difference (F – N)			
	Depth (μm)	Magnitude (μV)	99% CI (μV)	Depth (μm)	Time to Sink (ms)	Magnitude (μV^2^/μm^2^)	99% CI (μV^2^/μm^2^)
L2/3	100	146.83	88.22 208.29	340	98	−1.57 × 10^−3^	−3.16 × 10^−4^ −3.48 × 10^−3^
L4	20	155.75	96.74 214.86	20	62	−7.07 × 10^−4^	−1.73 × 10^−4^ −1.39 × 10^−3^
L5	−80	149.30	96.48 205.23	−80	75	−7.00 × 10^−4^	−1.40 × 10^−4^ −1.35 × 10^−3^
L6	−220	116.29	74.24 170.58	−360	84	−6.74 × 10^−4^	−2.43 × 10^−4^ −1.13 × 10^−3^

Layers of visual cortex are split into bands and the highest magnitude statistical difference within the layer is reported. Left, The depth, magnitude, and 99% confidence interval for the magnitude are reported. Right, The depth, time to the highest magnitude CSD sink, the magnitude of the CSD sink, and the 99% confidence interval for the CSD sink are reported. Statistics were calculated via a nonparametric hierarchical bootstrapping of 12 mice.

The local field potential is sensitive to volume conduction, so the measured VEP magnitude in one layer could be a consequence of activity in other layers. To isolate layer-specific changes, we used the CSD, wherein sinks represent flow of electrical current into cells. As expected, L4 is the site of the earliest and deepest sink for both familiar and novel stimuli (Fig. 3*G*). All layers display a deeper sink to familiar stimuli compared with novel stimuli. The earliest sink differences appear in L4 (Fig. 3*H*,*I*; Table 1, right; 99% CI = [−1.73 × 10^−4^ −1.39 × 10^−3^] µV^2^/µm^2^). Sink differences also appear in L2/3 (Fig. 3*H*,*I*; Table 1, right; 99% CI = [−3.16 × 10^−4^ −3.48 × 10^−3^] µV^2^/µm^2^), in L5 (Fig. 3*H*,*I*; Table 1, right; 99% CI = [−1.40 × 10^−4^ −1.35 × 10^−3^] µV^2^/µm^2^), and deep L6 (Fig. 3*H*,*I*; Table 1, right; 99% CI = [−2.43 × 10^−4^ −1.13 × 10^−3^] µV^2^/µm^2^). Thus, experience-dependent plasticity can be detected in superficial, middle, and deep layers of V1 using the CSD, indicating multiple potential sites of synaptic modification.

### Experience-dependent changes in the spectrum of LFP oscillations are seen at multiple depths

Oscillatory analysis of the continuous LFP signal can reveal consistent periodic structure in the electrical activity that reflects changing engagement of local neural circuitry. Because the portion of the recording containing the VEP violates second-order stationarity required for oscillatory analysis, we focused our analysis on the last 600 ms of each 1000-ms presentation when this violation no longer occurs. This approach is consistent with previous work (Chalk et al., 2010; Zhou et al., 2016; Hayden et al., 2021). Additionally, and in line with our previous work (Hayden et al., 2021), we normalized the raw spectrogram to the median spectrogram generated during black screen presentation. This normalized spectrum has units of decibel (dB).

**Figure 4.**
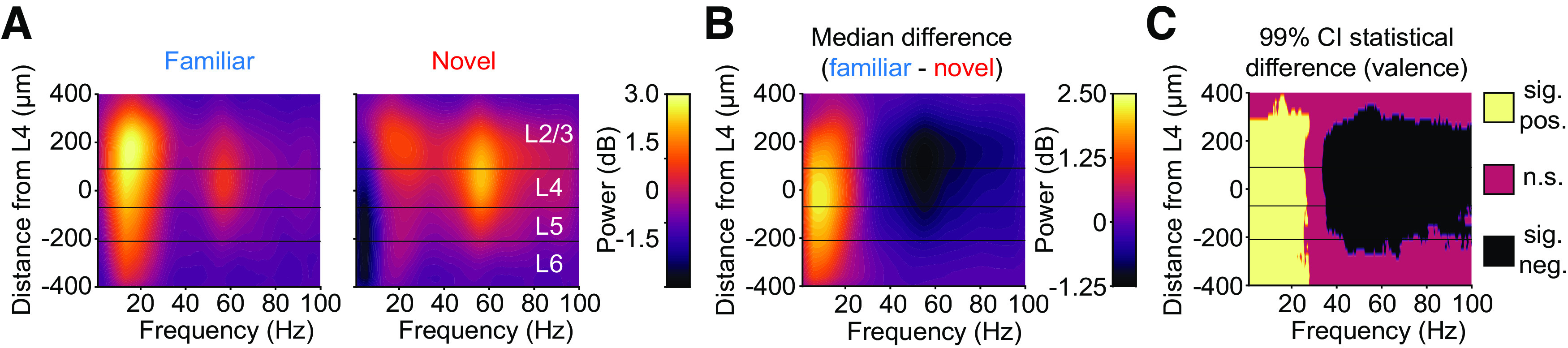
The normalized oscillatory power spectrum across multiple layers of V1 during familiar and novel stimulus viewing. ***A***, The laminar spectrum (normalized to black screen) during familiar and novel stimulus blocks (*n* = 12 mice). ***B***, Nonparametric hierarchical bootstrapping results for median normalized spectrum differences across layers. ***C***, Regions of the laminar VEP in ***B*** where bootstrapped 99% confidence interval does not include 0 (thus the difference is statistically significant). All plots use an average smoothing kernel that spans 10% of each axis. Three horizontal lines from top to bottom represent the boundaries between L2/3, L4, L5, and L6 as determined by inspection of the CSD.

We investigated whether the frequency composition of the LFP changes as a result of visual experience, as we have previously reported (Hayden et al., 2021). The normalized spectra during familiar and novel stimulus viewing showed the two expected findings: novel stimuli elicited more high-frequency power and familiar stimuli elicited more low-frequency power (Fig. 4*A*). The experience-dependent change in low-frequency power could be seen in all layers. The largest difference was in L4 (Fig. 4*B*,*C*; Table 2, left; 99% CI = [1.30 3.39] dB). However, L2/3 (Fig. 4*B*,*C*; Table 2, left; 99% CI = [1.06 3.05] dB), L5 (Fig. 4*B*,*C*; Table 2, left; 99% CI = [1.28 3.36] dB), and L6 (Fig. 4*B*,*C*; Table 2, left; 99% CI = [1.08 2.81] dB) also display this change. The experience-dependent change in high-frequency power could also be seen in most layers. The largest difference was evident in L2/3 (Fig. 4*B*,*C*; Table 2, right; 99% CI = [−0.66 −1.94] dB), but robust modulation was also observed in L4 (Fig. 4*B*,*C*; Table 2, right; 99% CI = [−0.63 −1.92] dB). In L5 the power difference was dampened (Fig. 4*B*,*C*; Table 2, right; 99% CI = [−0.43 −1.55] dB), and in L6 it was evident only toward the superficial boundary (Fig. 4*B*,*C*; Table 2, right; 99% CI = [−0.03 −0.90] dB). Thus, experience changes oscillatory activity in all layers, with modulation of low and high frequencies most prominent in superficial and middle layers, respectively.

**Table 2.** Familiarity changes the oscillatory activity in different layers of V1

	Low-frequency oscillations difference (F – N)				High-frequency oscillations difference (F – N)			
	Depth (μm)	Frequency (Hz)	Power (dB)	99% CI (dB)	Depth (μm)	Frequency (Hz)	Power (dB)	99% CI (dB)
L2/3	100	11.23	1.99	1.06 3.05	100	57.13	−1.25	−0.66 −1.94
L4	−60	10.01	2.33	1.30 3.39	80	57.13	−1.24	−0.63 −1.92
L5	−80	10.01	2.29	1.28 3.36	−80	57.37	−0.97	−0.43 −1.55
L6	−220	10.01	1.93	1.08 2.81	−220	57.37	−0.47	−0.03 −0.90

The depth, frequency, power, and 99% confidence interval for the power are reported for low-frequency oscillations (left) and high-frequency oscillations (right). Statistics were calculated via a nonparametric hierarchical bootstrapping of 12 mice.

### Familiarity decreases the average multiunit activity of superficial, Middle, and deep layers

**Figure 5.**
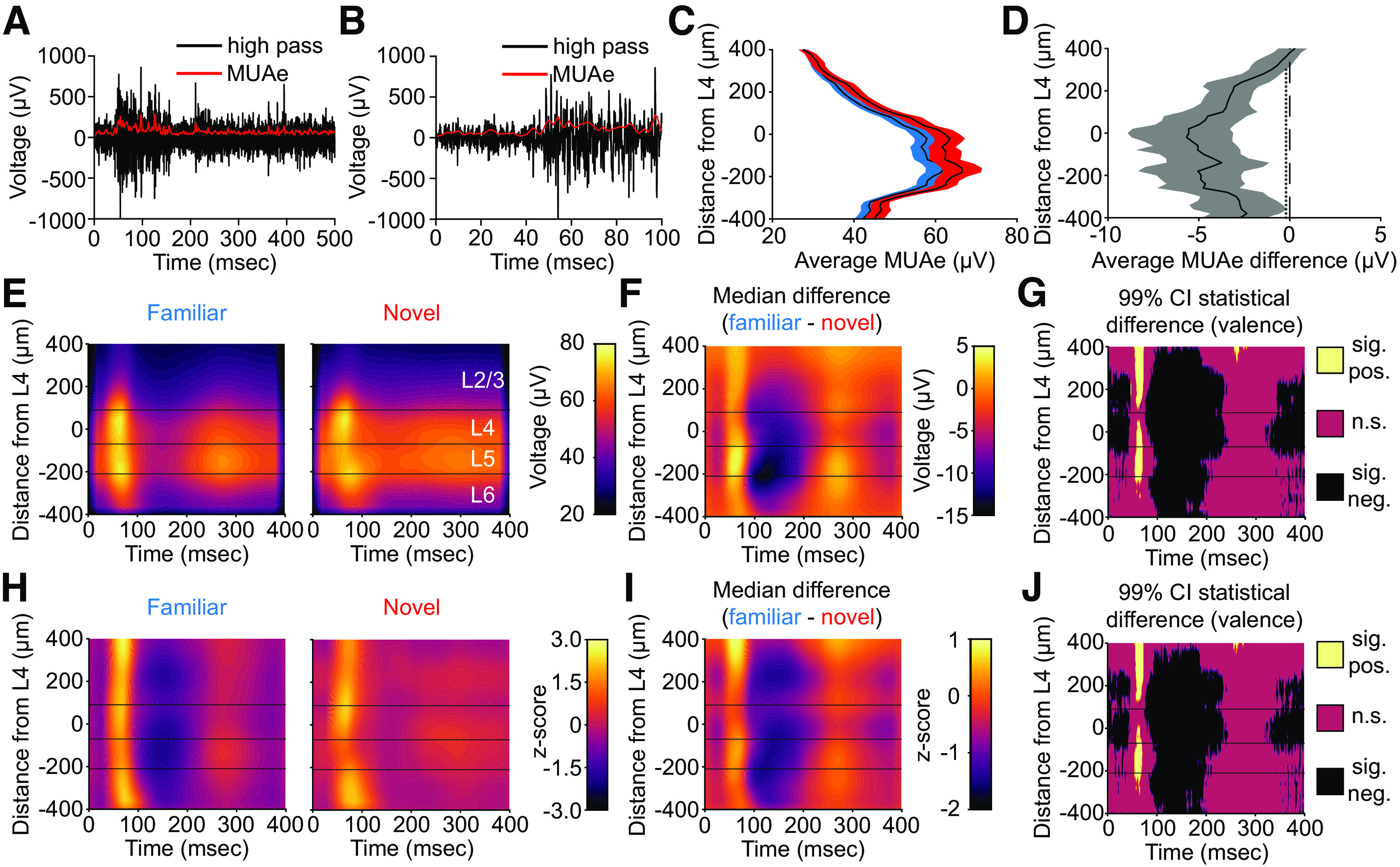
SRP expressed in the multiunit activity across multiple layers of V1. ***A***, ***B***, We calculated the envelope of multiunit activity (MUAe, see Materials and Methods) across layers of V1 (*n* = 12 mice). Example images show that MUAe closely tracks the raw high-pass filtered data. ***C***, The average evoked MUAe during familiar and novel stimulus blocks (collapsed from the first 400 ms of each phase reversal). Averaged activity is presented as mean ± SEM. ***D***, Nonparametric hierarchical bootstrapping results for average MUAe activity across layers. Data are presented as median bootstrap bounded by the 99% confidence intervals. Marks to the left of the vertical dashed 0 line indicate layers with a statistically significant F versus N difference (i.e., where the 99% bootstrapped difference does not contain 0). ***E***, The time course and amplitude of the average MUAe following phase reversals during familiar and novel stimulus blocks. ***F***, Nonparametric hierarchical bootstrapping results for median MUAe differences across layers. ***G***, Regions of the laminar MUAe in ***F*** whose bootstrapped 99% confidence interval does not include 0 (thus the difference is statistically significant). ***H–J***, As in ***E–G***, but for the z-scored (to gray screen) MUAe. All plots use an average smoothing kernel that spans 10% of each axis. Three horizontal lines represent the boundaries between L2/3, L4, L5, and L6 as determined by inspection of the CSD.

To investigate the spiking activity of V1, we calculated a value known as multiunit activity envelope (MUAe; Fig. 5*A*,*B*). MUAe provides an instantaneous measure of the number and size of action potentials of neurons in the vicinity of the electrode tip (Legatt et al., 1980; Brosch et al., 1995; Super and Roelfsema, 2005). This allowed us to use every channel in a recording, even if single units are not clearly isolatable, and it does not rely on an arbitrary threshold-crossing to assess multiunit activity. The raw MUAe has a large increase in deeper layers (Fig. 5*C*), likely corresponding to increased spiking activity in L5 (Senzai et al., 2019).

Previous studies have reported a decrease in average neural activity to familiar stimuli (T. Kim et al., 2020; Gao et al., 2021). We indeed find a small (5–10%), but significant, reduction in the average MUAe to familiar stimuli in L2/3 (Fig. 5*D*; Table 3, left; 99% CI = [−1.78 −5.93] µV), L4 (Fig. 5*D*; Table 3, left; 99% CI = [−3.15 −8.70] µV), L5 (Fig. 5*D*; Table 3, left; 99% CI = [−2.72 −8.44] µV), and L6 (Fig. 5*D*; Table 3, left; 99% CI = [−2.65 −7.06] µV). Thus, as with previous studies, familiarity reduces the average neural activity in V1.

**Table 3.** Familiarity changes the multiunit activity in different layers of V1

	Average MUAe difference (F – N)			Peak MUAe difference (F – N)				Trough MUAe difference (F – N)			
	Depth (μm)	Magnitude (μV)	99% CI (μV)	Depth (μm)	Time to Peak (ms)	Magnitude (μV)	99% CI (μV)	Depth (μm)	Time to Trough (ms)	Magnitude (μV)	99% CI (μV)
L2/3	100	−3.68	−1.78 −5.93	160	63	9.10	2.27 17.46	100	136	−9.86	−5.53 −14.46
L4	−20	−5.67	−3.15 −8.70	−60	65	10.34	4.23 16.41	−60	134	−11.99	−7.13 −16.81
L5	−80	−5.06	−2.72 −8.44	−160	63	17.62	2.45 45.01	−180	144	−17.09	−8.54 −29.52
L6	−220	−4.90	−2.65 −7.06	−220	65	12.84	2.10 22.39	−240	103	−16.84	−8.82 −28.31

(Left) The depth, magnitude, and 99% confidence interval for the magnitude of the average MUAe are reported. The depth, time, MUAe magnitude, and 99% confidence interval for the MUAe magnitude are reported for the peak firing rate (center) and the trough firing rate (right). Statistics were calculated via a nonparametric hierarchical bootstrapping of 12 mice.

However, while the average neural activity reduces in response to stimulus experience, previous results from our lab have shown an increase in peak activity to familiar stimuli (Cooke et al., 2015). Thus, we next looked at the event related MUAe (Fig. 5*E*). Visual inspection reveals a stereotyped difference between familiar and novel stimuli across all layers. This pattern featured a short-latency increase in peak activity followed by a relative quiescent period between phase reversals of the familiar stimulus, as compared with the more consistent activity observed during presentation of the novel stimulus (Fig. 5*F*,*G*). To quantify this, we report the difference in average maximum activity (i.e., peak) within the first 100 ms of the phase-reversal. We also report the difference in the minimum activity (i.e., trough) within the subsequent 100 ms. The largest peak difference is in L5 (Fig. 5*F*,*G*; Table 3, center; 99% CI = [2.45 45.01] µV). Peak differences are also seen in L2/3 (Fig. 5*F*,*G*; Table 3, center; 99% CI = [2.27 17.46] µV), L4 (Fig. 5*F*,*G*; Table 3, center; 99% CI = [4.23 16.41] µV), and superficial L6 (Fig. 5*F*,*G*; Table 3, center; 99% CI = [2.10 22.39] µV). Relative to novel, the familiar stimulus elicits a period of quiescence that is most prominent in L5 (Fig. 5*F*,*G*; Table 3, right; 99% CI = [−8.54 −29.52] µV) and superficial L6 (Fig. 5*F*,*G*; Table 3, right; 99% CI = [−8.82 −28.31] µV), but is also evident in L2/3 (Fig. 5*F*,*G*; Table 3, right; 99% CI = [−5.53 −14.46] µV) and L4 (Fig. 5*F*,*G*; Table 3, right; 99% CI = [−7.13 −16.81] µV).

To aid visualization, we z-scored the stimulus-evoked activity for both familiar and novel stimuli to the same average and SD values (see Materials and Methods; Fig. 5*H*). This allowed us to make direct comparisons between familiar and novel stimuli in all cortical layers while preserving an optimal range for the color scale used in each plot. These values are unitless. The z-scored MUAe difference for familiar and novel data showed the expected peak activity followed by a quiescent period (Fig. 5*I*,*J*). A z-scored peak difference exists in all layers (Fig. 5*I*,*J*; Table 4, left; L2/3: 99% CI = [1.21 3.39]; L4: 99% CI = [0.47 2.05]; L5: 99% CI = [0.78 2.65]; L6: 99% CI = [0.24 2.47]). Additionally, a z-scored trough difference exists in all layers (Fig. 5*I*,*J*; Table 4, right; L2/3: 99% CI = [−0.98 −2.71]; L4: 99% CI = [−0.89 −2.29]; L5: 99% CI = [−0.82 −2.87]; L6: 99% CI = [−1.02 −2.52]). Thus, long-term stimulus familiarity increases the peak activity in superficial, middle, and deep layers of V1 before a period of quiescence that is potentially attributable to the rapid recruitment of strong feedback inhibition.

**Table 4.** Familiarity changes the normalized multiunit activity in different layers of V1

	Peak MUAe z-scored difference (F – N)				Trough MUAe z-scored difference (F – N)			
	Depth (μm)	Time to peak (ms)	Magnitude (a.u.)	99% CI (a.u.)	Depth (μm)	Time to trough (ms)	Magnitude (a.u.)	99% CI (dB)
L2/3	400	66	2.31	1.21 3.39	220	142	−1.79	−0.98 −2.71
L4	−60	65	1.28	0.47 2.05	−60	134	−1.59	−0.89 −2.29
L5	−120	65	1.68	0.78 2.65	−180	144	−1.78	−0.82 −2.87
L6	−220	65	1.46	0.24 2.47	−220	136	−1.72	−1.02 −2.52

The depth, time, z-scored MUAe magnitude, and 99% confidence interval for the z-scored MUAe magnitude are reported for the peak firing rate (left) and the trough firing rate (right). Statistics were calculated via a nonparametric hierarchical bootstrapping of 12 mice.

**Figure 6.**
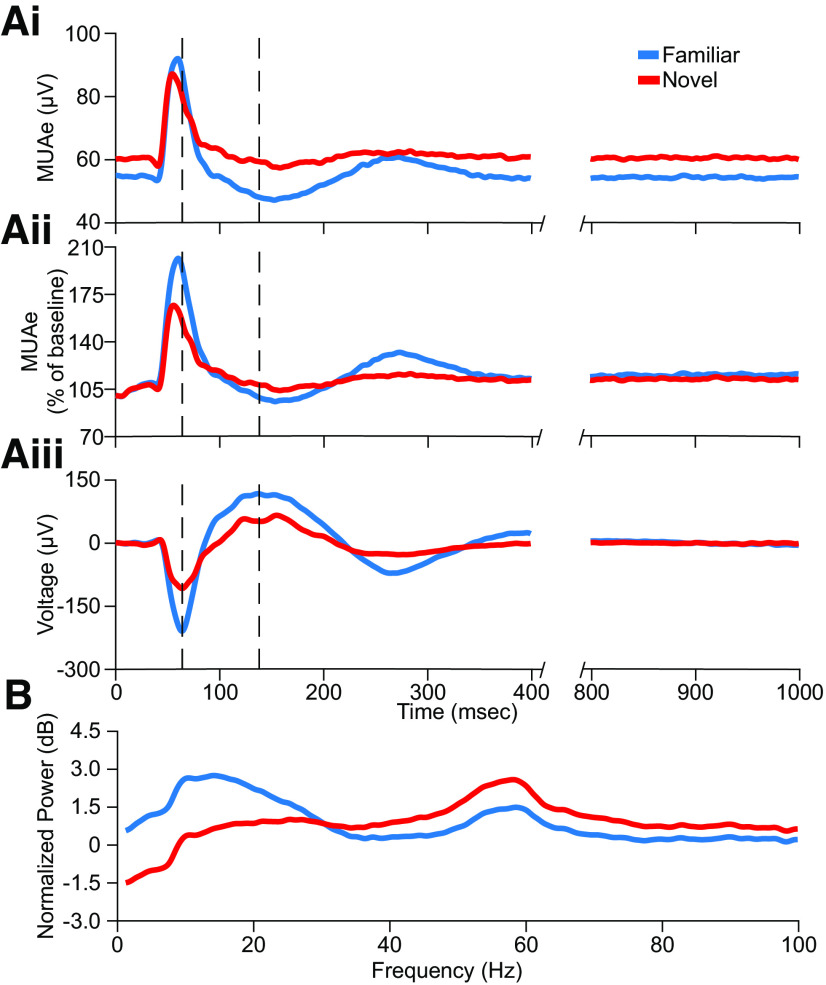
Comparison of SRP expressed in the multiunit activity, VEP, and normalized spectrum in L4 of V1. ***Ai***, The average MUAe during familiar and novel stimulus blocks for L4 (replotted from Fig. 5*E*). Familiar stimuli induce lower overall activity, but a phase reversal causes an increase in peak activity compared to novel that is followed by a prolonged period of quiescence. ***Aii***, As in ***Ai*** but normalized to the average prephase-reversal baseline. ***Aiii***, The average VEP during familiar and novel stimulus blocks for L4 (replotted from Fig. 3*D*). Familiar stimuli induce a larger peak-to-peak VEP magnitude. Dashed vertical lines in ***Ai***, ***Aii***, and ***Aiii*** are the timepoints where the familiar VEP has the maximal negativity and positivity, respectively. ***B***, The average normalized spectrum during familiar and novel stimulus blocks for L4 (replotted from Fig. 4*A*). This spectrum is calculated using the local field potential data between 400 and 1000 ms postphase-reversal-onset to prevent the contamination of the spectral estimate by the VEP response (see Materials and Methods). Familiar stimuli induce an increase in the low frequency power and a decrease in high frequency power.

In Figure 6, we compare three different measures of learned stimulus familiarity recorded simultaneously in L4. The MUAe shows both the augmented peak firing and the downward “DC shift” in activity between phase reversals (Fig. 6*Ai*). To compare the unit data with VEPs, we normalized the MUAe to the prephase-reversal baseline (Fig. 6*Aii*). Normalization clearly shows how the firing rate change from baseline is dramatically augmented when familiar stimuli are phase reversed compared with novel. The peak and trough of the multiunit activity closely align with the negative-going and the positive-going component of the VEP, respectively (Fig. 6*Aiii*). For such a simple measure, the L4 VEP is remarkably sensitive and information rich when it comes to detecting experience-dependent changes in cortical information processing. Moreover, the same L4 VEP recording electrode detects changes in activity between phase reversals as revealed by oscillatory activity in the LFP (Fig. 6*B*).

## Discussion

Despite abundant evidence supporting the hypothesis that potentiation of thalamocortical synapses is the molecular basis for SRP (Frenkel et al., 2006; Cooke and Bear, 2010), it is now very clear that this does not occur on excitatory neurons in L4 (Montgomery et al., 2021). Indeed, recent findings indicate that SRP expressed as VEP potentiation in L4 is most parsimoniously explained by an experience-dependent reduction in inhibition mediated by PV+ interneurons in this layer (Kaplan et al., 2016; Hayden et al., 2021). Our aims in the current study were therefore to reexamine participation of excitatory neurons in the mechanisms of SRP, with a particular focus on L6 cells known to be a direct target of thalamocortical input, and to perform a laminar analysis of how SRP is expressed across all layers of V1.

To accomplish our first aim, we deleted NMDARs, first from excitatory neurons across all layers, and then from a specific population of L6 projection neurons for which we had genetic access. These *Ntsr1-*expressing L6 neurons were of particular interest, first, because they have been shown to strongly modulate inhibition in L4 through a population of PV+ neurons with translaminar axonal projections, second, because they project back to the thalamus where they can modulate feedforward activation of V1 (Olsen et al., 2012; Bortone et al., 2014; J. Kim et al., 2014; Guo et al., 2017; Voigts et al., 2020), and third, because they, like SRP, are highly orientation-selective (Vélez-Fort et al., 2014). With respect to SRP of VEPs in L4, knock-out of NMDARs only within *Ntsr1-*expressing L6 neurons phenocopied the loss of NMDARs from excitatory neurons across all layers of V1, supporting the conclusion that these L6 neurons are of particular significance for expression of SRP in L4. This finding is consistent with previous work showing ablation or molecular manipulation of L6 neurons in higher order visual cortex strongly influences object recognition memory (López-Aranda et al., 2009), and stands in stark contrast to what was observed when NMDARs were deleted in L4 principal neurons (Fong et al., 2020). Interpretation of the SRP phenotype observed after knocking out NMDARs in L6 is not straightforward, however. First, loss of NMDARs within *Ntsr1*-expressing L6 neurons could have reduced orientation selectivity, thus confounding our measurements of familiar and novel stimuli. However, the familiar and novel stimuli were orthogonal orientations, meaning that discrimination was as unchallenging as it could possibly be. Even if orientation selectivity was compromised by the loss of NMDARs, this change alone would not be expected to impact plasticity across days induced by a single orientation (Fig. 2*E–G*). Thus, there is a deficit in SRP after our cell type-specific NMDAR knock-outs that cannot be accounted for by a loss of orientation selectivity. Second, had loss of NMDARs only blocked experience dependent synaptic plasticity in L6, we would expect to observe no baseline change in VEP amplitude as well as no SRP. However, instead we observed a clear increase in VEP magnitude at baseline in response to stimuli the mice had not previously experienced. Thus, the diminished effect of subsequent visual experience could either reflect an impairment in NMDAR-dependent feedforward synaptic plasticity (e.g., impaired LTP; Cooke and Bear, 2010), a failure to consolidate SRP via corticothalamic feedback (Durkin et al., 2017), or the partial occlusion of SRP caused by reduced activation of these L6 neurons in the absence of NMDARs and a consequent reduction in excitatory drive from these neurons onto PV+ neurons providing inhibition in layer 4. Third, it is difficult to say whether Ntsr1-GluN1 KO animals are failing to potentiate to familiarity, failing to suppress or modulate cortical response with respect to day 1 novelty, or some combination of both. More work is needed to parse these possible explanations, but we can confidently conclude based on available data that L6 neurons contribute to a circuit that plays a critical role in the expression of SRP in L4 VEPs.

The profound influence of a molecular manipulation of L6 on plasticity measured in L4 underscored the need to develop a more thorough description of SRP across all layers of V1. This was accomplished using translaminar multichannel electrodes which allowed analysis of VEPs and spiking activity elicited by phase reversals of novel and familiar stimuli, as well as changes in oscillatory activity in the LFP between phase reversals. Across superficial, middle, and deep cortical layers we observed that VEP magnitudes and the spectral content of V1 activity elicited by familiar and novel stimuli were strikingly different, extending previous findings restricted to L4 (Frenkel et al., 2006; Cooke and Bear, 2010; Cooke et al., 2015; Kaplan et al., 2016; Fong et al., 2020; T. Kim et al., 2020; Hayden et al., 2021). Other than responses measured near the cortical surface, VEP magnitudes were elevated in response to phase reversals of familiar stimuli compared with novel across all layers, notably including L6 (Fig. 3). CSD analysis revealed the expected translaminar progression of information flow in V1 (Mitzdorf, 1985; Aizenman et al., 1996) and confirmed augmentation of both short-latency and long-latency current sinks in all layers except deep L5. Similarly, the signature of stimulus familiarity in the LFP oscillations measured between phase reversals was clearly present in all layers (Fig. 4). However, whereas the enhanced power in the low-frequency (∼15 Hz) band occurred at all depths, reduced power in the high-frequency γ band (∼65 Hz) appeared to be confined to the middle layers, centered on L4. This finding is of interest, as decreased γ is associated with reduced recruitment of PV+ interneurons (Chen et al., 2017; Veit et al., 2017), which likely accounts for VEP potentiation in L4 (Kaplan et al., 2016).

We found comparable modulation of multiunit activity in superficial, middle, and deep layers of V1 by familiar visual stimuli that is best described as a temporal redistribution of cell firing (Fig. 5). In all layers, there was an increase in the multiunit peak firing in response to phase reversals of a familiar stimulus compared with a novel stimulus. However, this averaged phasic increase in firing rate was quickly followed by a prolonged period of quiescence between phase reversals, leading to an overall reduction in population activity during familiar stimulus viewing compared with novel. This finding is consistent with observations using intracellular recordings from single neurons in superficial V1, showing a reduction in average firing rate to sustained viewing of familiar stimuli (Gao et al., 2021). It is of interest to compare our electrophysiological observations with previous studies using two-photon calcium imaging to estimate cellular activity. Imaging of pyramidal cells in superficial layers and L4 have shown consistently a reduction in averaged calcium sensor fluorescence in response to familiar stimuli in comparison to novel stimuli (Kato et al., 2015; Makino and Komiyama, 2015; T. Kim et al., 2020). The study by T. Kim et al. (2020) in L4 is particularly relevant, as it used the same SRP protocol we used in the current study. We think it is likely that the fleeting increase in firing after each phase reversal, lasting tens of milliseconds, was likely missed because of the relatively poor temporal resolution of the imaging method. In any case, these differences highlight how use of phase reversing stimuli and electrophysiology to probe modifications of V1 that accompany visual learning can yield novel mechanistic insights that could be missed using other stimulation and recording methods (e.g., drifting gratings and calcium imaging). Without getting too speculative about the precise circuit elements involved, our data are generally consistent with a model in which learning occurs by enhancement of net feedforward thalamocortical excitation (revealed by the phasic response potentiation) that, in turn, recruits increased polysynaptic feedback inhibition that quenches the activity between phase reversals. In this model, the reduced tonic activation of cortex during familiar stimulus viewing is a consequence of a net increase in inhibitory tone rather than a long-term depression of excitatory synaptic transmission.

In conclusion, in addition to showing how profoundly stimulus familiarity influences activity across all layers of mouse V1, we have identified a surprising influence of L6 excitatory neurons on producing pronounced differences in the response of L4 to familiar and novel stimuli. Further work will be required to determine whether this influence occurs through a PV+ inhibitory intermediary as suggested by our previous observations (Kaplan et al., 2016; Hayden et al., 2021) and, if so, whether these intermediaries reside within L6 (Bortone et al., 2014; Frandolig et al., 2019) or in more superficial layers (J. Kim et al., 2014). In addition, further work will be required to understand whether the influence of *Ntsr1*-expressing L6 neurons occurs via their characteristic feedback to the primary sensory thalamus. It has been shown that feedback from L6 neurons to the dorsal thalamus produces both direct excitation of the primary relay nucleus of the thalamus, in this case the dLGN, as well as inhibition resulting from disynaptic feedback via the all-inhibitory thalamic reticular nucleus (TRN; Denman and Contreras, 2015). The balance of excitatory and inhibitory feedback to the dorsal thalamus and, in turn, the direction of influence on thalamo-recipient cortical layers can be flipped by plasticity occurring within this feedback circuitry (Crandall et al., 2015). Thus, it remains to be seen whether the influence of *Ntsr1*-expressing L6 neurons on layer four neural activity occurs primarily via intracortical connectivity, or via key thalamic circuits, or through some mixture of both. Either way, the activity of these L6 neurons may control the important trade-off between stimulus detection and perception of stimulus features (Guo et al., 2017). Thus, the switch in the mode of activity in canonical cortical circuitry produced by L6 CT neurons potentially serves the primary function of habituation by limiting the influence of familiar sensory experience on attention and energy use, while enhancing vigilance for unexpected changes in the environment.
